# A comparative study of two methods to predict the incidence of hepatitis B in Guangxi, China

**DOI:** 10.1371/journal.pone.0234660

**Published:** 2020-06-24

**Authors:** Yanling Zheng, Liping Zhang, XiXun Zhu, Gang Guo

**Affiliations:** 1 College of Medical Engineering and Technology, Xinjiang Medical University, Urumqi, People’s Republic of China; 2 School of Computer Engineering, Jingchu University of Technology, Jingmen, People’s Republic of China; 3 State Key Laboratory of Pathogenesis, Prevention and Treatment of High Incidence Diseases in Central Asia, Clinical Medicine Institute, the First Affiliated Hospital of Xinjiang Medical University, Urumqi, People’s Republic of China; South China University of Technology, CHINA

## Abstract

In recent years, the incidence of hepatitis B (HB) in Guangxi is higher than that of the national level; it has been increasing, so it is urgent to do a good predictive research of HB incidence, which can help analyze the early warning of hepatitis B in Guangxi, China. In the study, the feasibility of predicting HB incidence in Guangxi by autoregressive integrated moving average (ARIMA) model method and Elman neural network (ElmanNN) method was discussed respectively, and the prediction accuracy of the two models was compared. Finally, we established the ARIMA (0, 1, 1) model and ElmanNN with 8 neurons. Both ARIMA (0, 1, 1) model and ElmanNN model had good performance, and their prediction accuracy were high. The fitting and prediction root-mean-square error (RMSE) and mean absolute error (MAE) of ElmanNN were smaller than those of ARIMA (0, 1, 1) model, which indicated that ElmanNN was superior to ARIMA (0, 1, 1) model in predicting the incidence of hepatitis B in Guangxi. Based on the ElmanNN, the HB incidence from September 2019 to December 2020 in Guangxi was predicted, the predicted results showed that the incidence of HB in 2020 was slightly higher than that in 2019 and the change trend was similar to that in 2019, for 2021 and beyond, the ElmanNN model could be used to continue the predictive analysis.

## Introduction

Hepatitis B (HB) is a chronic infectious disease caused by HB virus infection in liver tissue. If HB is not treated in time, a small number of patients with liver tissue will further appear inflammatory aggravation, liver cirrhosis, liver cancer [1]. At present, there are about 300 million people living with chronic HB virus (including chronic HB patients and HB virus carriers), mainly in East Asia, South Asia, sub-Africa and other regions [2]. Due to the large number of people living with chronic HB virus, it has become an important public health problem in the world. China is the country with the heaviest burden of HB infection. At present, there are about 100 million chronic HB virus infections in China. However, the new cases of liver cancer in China account for about 59% of the world every year, most of which are caused by hepatitis B virus infection and hepatitis C virus infection [3,4]. Therefore, the current situation of HB prevention and control in China is still very serious.

In order to help the prevention and control of hepatitis B, research from different perspectives should be done, such as the early warning research, the analysis of socio-economic factor, demographic factors and meteorological environment factors of affecting HB, etc, among them, reasonable prediction and early warning links can help to do a good job of resource demand planning in advance, so as to formulate the corresponding prevention and control preparations.

The prediction analysis includes qualitative prediction and quantitative prediction, qualitative prediction is fuzzy and usually used for descriptive expression, while quantitative prediction is more precise and usually used to predict more accurate future situation by data analysis. At present, quantitative prediction is widely used in various fields, and the model methods used are various, such as: neural network models were used to predict crash frequency [5,6], and Bayesian spatial generalized ordered logit model was developed for predicting the crash severity [7]; the integrated approach of belief rule base and deep learning was used to predict air pollution [8]; machine-learning techniques was used to accurately predict battery life [9]; regression model was used to energy performance of a net-zero energy building [10], etc, the results of these studies can help early analysis for the future situation of these research field, so as to do the planning work well in advance and reduce loss. In recent years, many mathematical model methods were used to predict the incidence of infectious diseases, such as linear model [11,12], dynamics model [13,14], grey model [15], time series ARIMA model, neural network model, and so on. Since the time series of infectious diseases often have the characteristics of trend and randomness, ARIMA model and neural network model can capture the regularity of such data well, so they were most widely used and obtained good prediction performance and high prediction accuracy [16–24].

In China, the incidence of HB in Guangxi Province is higher than that of the national level, and it has an upward trend in recent years, so the task of prevention and control of HB in Guangxi Province is very serious. This study attempts to help analyze the early warning of hepatitis B. Based on the data of HB incidence from January 2012 to August 2019 in Guangxi, we studied the feasibility of predicting HB incidence in Guangxi by ARIMA model and Elman neural network model, and compared the prediction effects of the two models and did predictive analysis, so as to provide scientific reference for the prevention and control of HB in Guangxi.

## Materials and methods

### Study area and data sources

Guangxi is located in the south of China, between 20°54' to 26°24' N and 104°26' to 112°04' E. It covers an area of 236,700 square kilometers and has a population of 49.26 million at the end of 2018. This study collected the data of hepatitis B cases in Guangxi and the population data of Guangxi from January 2012 to August 2019. The reported case data of hepatitis B (HB) in Guangxi came from the website of Health Commission of Guangxi, China, and the population data came from Guangxi Bureau of Statistics. The incidence data of HB were calculated by using the case data and population data (see S1 File). Matlab2016b and Eviews7 were used for data analysis.

### ARIMA model

ARIMA (p, d, q) model is an important time series analysis and prediction model, which is also called Autoregressive Integrated Moving Average Model [25,26]. Because the model can capture the trend and randomness of data, it is widely used in the prediction of infectious diseases, and has achieved good prediction results, such as, Wang et al. [16] found that ARIMA model could predict the morbidity of influenza in Ningbo, China, 2006–2014, successfully; Shen et al. [18] analyzed that ARIMA model was successful in predicting hemorrhagic fever with renal syndrome in China; Anokye et al. [21] found that ARIMA model had good performance in predicting malaria incidence; etc [17,19,20].

The ARIMA (p, d, q) model is established on the basis of stationary time series, so the stationarity of time series is an important prerequisite for modeling. The Augmented Dickey-Fuller (ADF) unit root test method is generally used to test the stability of time series. If the significant level P is less than 0.05, the data is stable, otherwise, the time series can also be stable through some operations (for example, logarithm, difference, if the n order difference is done, d = n). When d is 1, its mathematical expression is as follows

$$y_{t}=\phi_{1}y_{t-1}+\phi_{2}y_{t-2}+\cdots +\phi_{p}y_{t-p}+\varepsilon_{t}-\theta_{1}\varepsilon_{t-1}-\theta_{2}\varepsilon_{t-2}-\cdots -\theta_{q}\varepsilon_{t-q},$$

where, X_t_ is the incidence of t time, *y*_*t*_ = X_t_-X_t-1_, *ϕ*_*i*_,*i* = 1,2,⋯,*p* and *θ*_*i*_,*i* = 1,2,⋯,*q* are parameters, p is the autoregressive order and q is the moving average order.

The modeling process includes model identification, parameter estimation, hypothesis testing and residual analysis. Firstly, the ACF and PACF diagrams of stationary data are made to determine the possible values of p and q, and then the parameter estimation and parameter hypothesis test are made. Next, the residual analysis of the model passed the parameter test is carried out, if the self-correlation and the partial correlation coefficient of the model residue are within the standard deviation of two times, then the model residual is white noise, which indicates that the established model has good performance and can be used for prediction and analysis.

### Elman neural network model [27,28]

Elman neural network (ElmanNN) was first proposed by J. L. Elman in 1990 to solve the problem of speech processing. It is a typical local regression network. Elman network can be regarded as a recurrent neural network with local memory unit and local feedback connection. It is a dynamic feedback network, which can feedback, store and use the output information of the past time. It can not only realize the modeling of static system, but also realize the mapping of dynamic system and directly reflect the dynamic characteristics of the system, it is better than propagation neural network in computing power and network stability. Elman neural network is very suitable for time-series prediction, in many fields of prediction research; it has achieved many successful results, such as Liu et al. [29] did the analysis of wind speed prediction using ElmanNN; Ismael et al. [30] predicted the data consumption for power demands by ElmanNN successfully; Li et al. [31] did the prediction of Urban Rail Transit Sectional Passenger Flow Based on ElmanNN successfully; etc [32–34].

It consists of input layer, hidden layer, bearing layer and output layer. The mathematical expressions of Elman network structure used in this study are as follows:

$$x(k)=f(w^{1}x_{c}(k)+w^{2}u(k-1)),$$

$$x_{c}(k)=x(k-1),$$

$$y(k)=g(w^{3}x(k)).$$

Where, *y*,*x*,*u* and *x*_*c*_ represent *m*-dimensional output vector, *n*-dimensional implicit layer node vector, *r*-dimensional input vector and *n*-dimensional feedback state vector, respectively. *w*^1^,*w*^2^ and *w*^3^, respectively, represent the connection weight from the receiving layer to the hidden layer, the connection weight from the input layer to the hidden layer, and the connection weight from the hidden layer to the output layer. *g*(*w*^3^*x*(*k*)) is the transfer function of the output neuron, which is the linear combination of the hidden layer output. *f*(*w*^1^*x*_*c*_(*k*)+*w*^2^*u*(*k*−1)) is the transfer function of hidden layer neurons.

In general, if the number of neurons in the hidden layer is too small, the network will not be able to establish a complex mapping relationship, so that the network cannot be trained, or cannot identify the previous samples, and the fault tolerance is poor, if the number of neurons is too large, then the network learning time is too long, and the error is uncertain the smallest. In this paper, the optimal number of neurons is selected from 1 to 20 according to the value of training sample and its fitting mean square error (MSE), and the number of neurons corresponding to the minimum MSE value is the best number of neurons. In this study, the Elman network establishment process is shown in S2 File.

### Model comparison

Root-mean-square error (RMSE) and mean absolute error (MAE) are used to compare the performance of two models, the smaller the two values, the better the model.

## Results

### Establishment of ARIMA model

**Fig 1:**
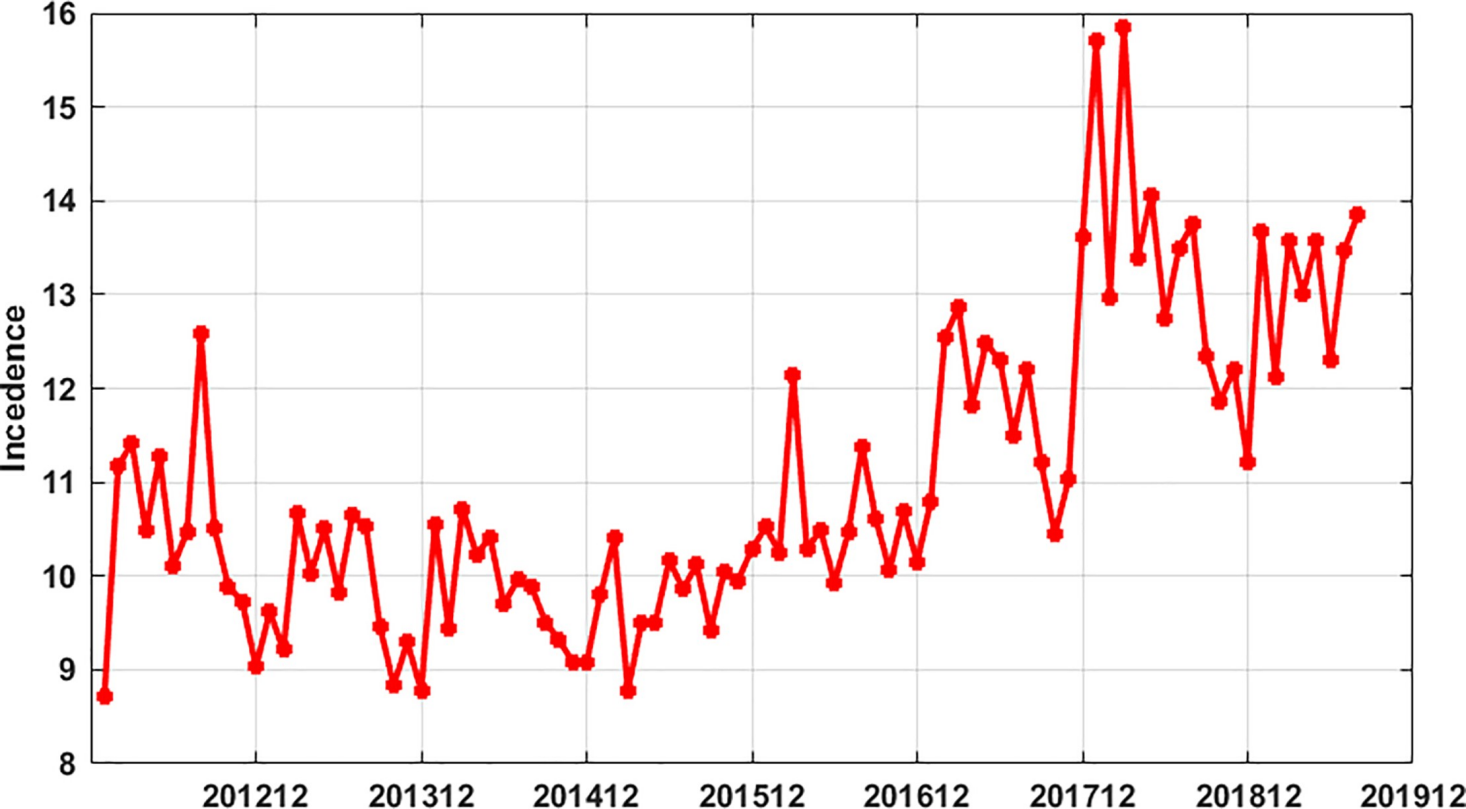
Incidence of hepatitis B in Guangxi from January 2012 to August 2019.

From January 2012 to August 2019, 486983 cases were reported in Guangxi and 183 cases died, from 2016 to 2019, the incidence of hepatitis B in Guangxi showed an upward trend, especially in 2018. See Fig 1.

**Fig 2:**
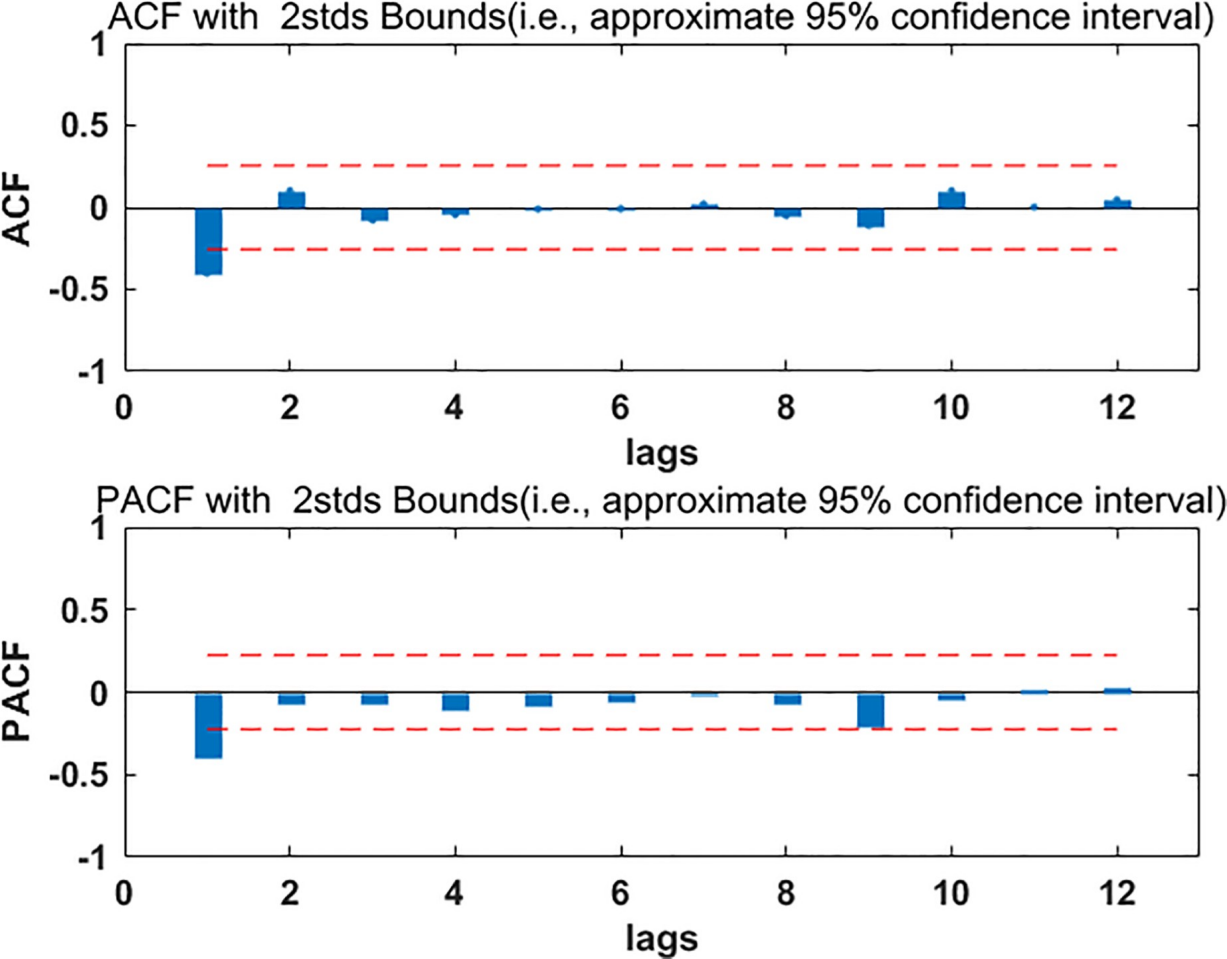
The ACF and PACF diagrams of stationary data. ACF = autocorrelation function, PACF = partial autocorrelation function.

**Fig 3:**
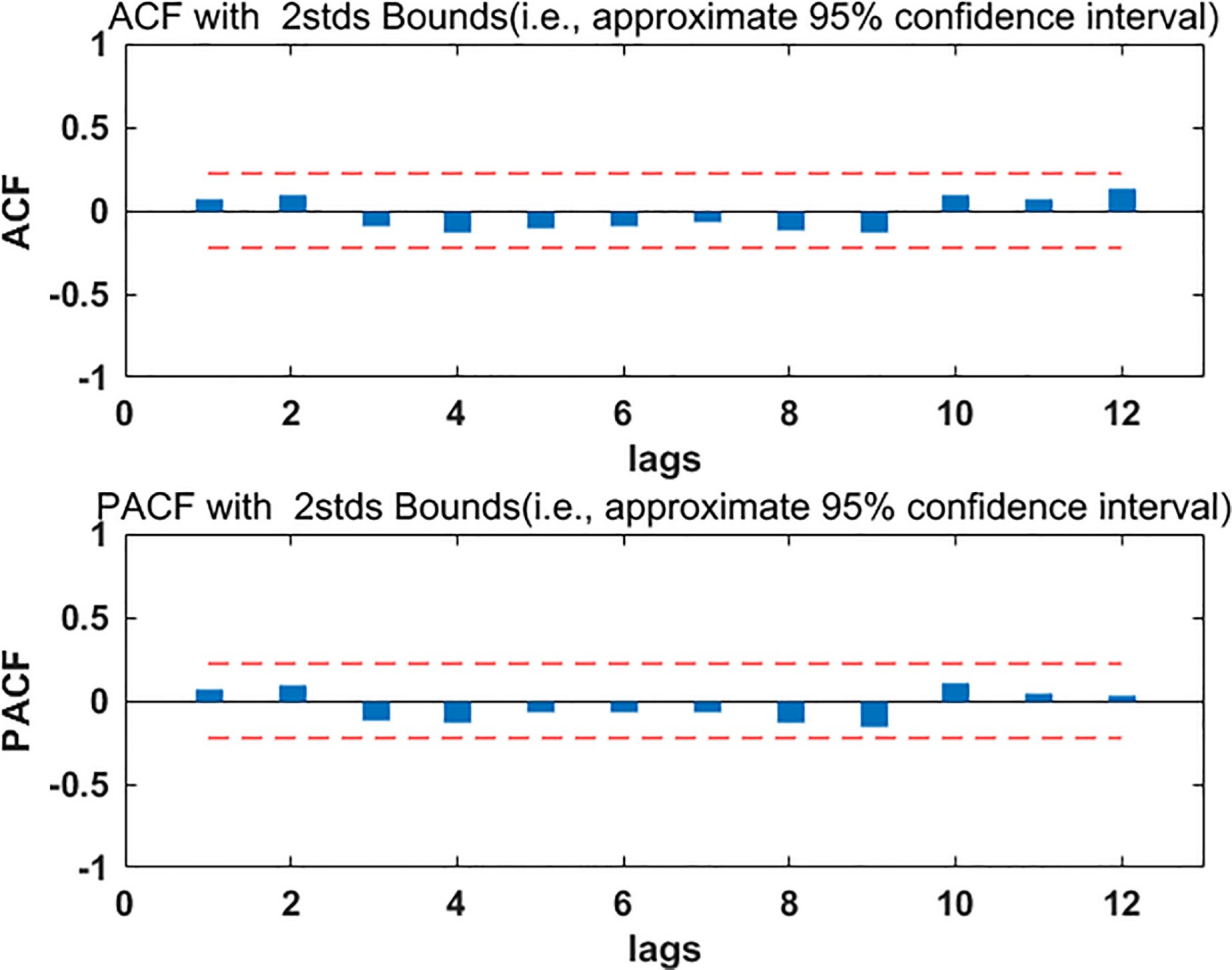
The ACF and PACF diagrams of residual errors of ARIMA (0, 1, 1) model. ACF = autocorrelation function, PACF = partial autocorrelation function.

The data from January 2012 to August 2019 were divided into two parts, the part from January 2012 to December 2018 was used to build the model, and the part from January 2019 to August 2019 was used to test the model. The ARIMA model requires the data to be stable. Firstly, the stability of the modeling part of the data was checked, the ADF test result P was more than 0.05, which indicated that the data were not stable. In order to make the data stable, the first order difference of the data was done, and then the ADF test was performed. The test results were shown in Table 1. P was less than 0.05, which indicated that the data were stable after the first order difference, and d equaled 1. Draw ACF and PACF diagrams for stationary data, look at Fig 2, from which we determined p, q take 0 or 1, so the tentative models were ARIMA (1, 1, 1), ARIMA (0, 1) and ARIMA (1, 1, 0). The least square method was used to test the parameters of the three models, and only the ARIMA (0, 1, 1) model passed the test, then the residual analysis of the ARIMA (0, 1, 1) model was carried out. From the residual ACF and PACF graph (Fig 3), it can be seen that the self-correlation and the partial correlation coefficient of the model residue were within the standard deviation of two times, which indicated that the model residual was white noise, so, the ARIMA (0, 1, 1) was feasible. Based on this model, the incidence data of HB in Guangxi from January 2012 to December 2018 were fitted. The fitting RMSE was 0.95, the fitting MAE was 0.7; the incidence data of HB in Guangxi from January 2019 to August 2018 were predicted, the prediction RMSE was 0.94 and the prediction MAE was 0.81.

**Table 1:** The ADF test of the modeling data.

t-Statistic			P
Augmented Dickey-Fuller test statistic		-14.17	0.0001
**Test critical values:**	**1**% **level**	**-3.51**	
	**5**% **level**	**-2.9**	
	**10**% **level**	**-2.59**	

### Establishment of ElmanNN model

**Fig 4:**
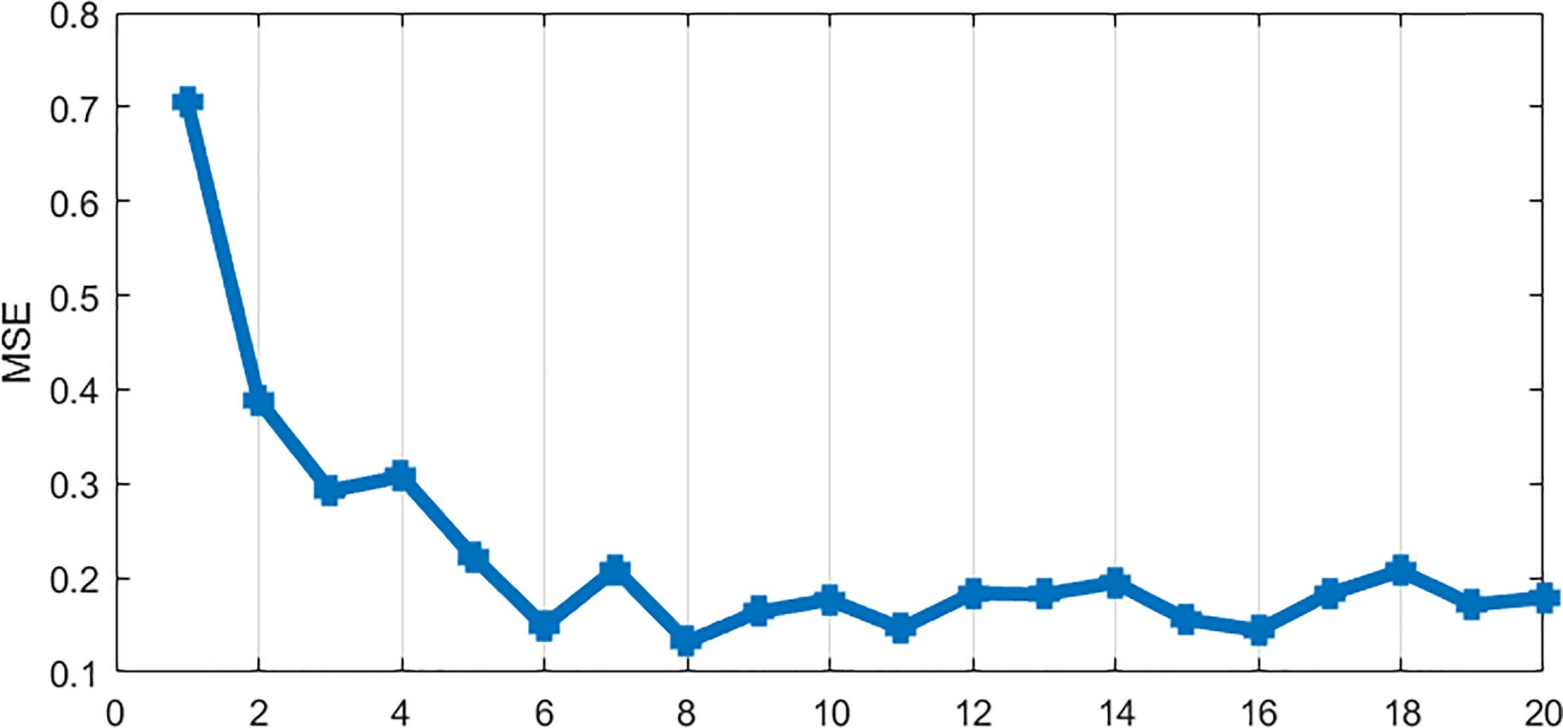
The numbers of neurons in ElmanNN and corresponding MSE values.

**Fig 5:**
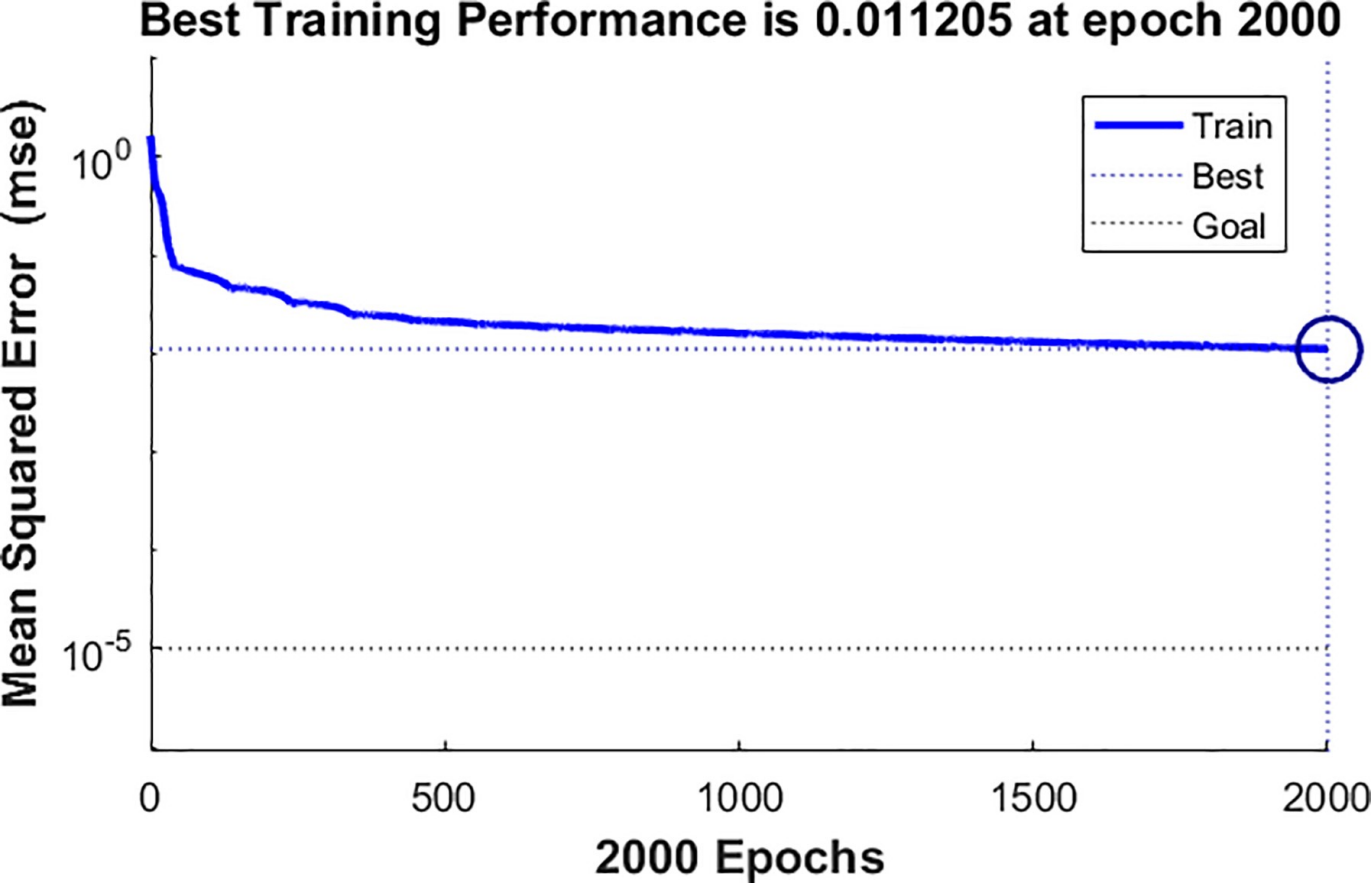
The error drop curve of Elman network.

After careful observation of Fig 1, we could find that there was a certain similarity in the sequence change map of HB incidence each year; therefore, we created twelve time-lagged variables as input features. The incidence data of hepatitis B in Guangxi were processed as follows input and output matrix,

$$input\;matrix=\left[\begin{array}{l} x_{1}\;x_{2}\;\cdots \;x_{i}\\ x_{2}\;x_{3}\;\cdots \;x_{i+1}\\ \;\cdots \cdots \cdots \\ x_{N}\;x_{N+1}\cdots \\ x_{N+1}\;x_{N+2}\cdots \end{array}\right],\mathrm{out}\;put\;matrix=\left[\begin{array}{l} x_{N+1}\\ x_{N+2}\\ \cdots \\ x_{N+m} \end{array}\right]$$

where, *x*_i_ represents the incidence of HB at time *i*, N = 12. m is the number of input variables, and the first input variable is (*x*_1,_ *x*_2,………,_ *x*_*i*_), the second input variable is (*x*_2,_ *x*_3,………,_ *x*_*i*+1_), and so on. In this study, m was 12, and the matlab cyclic structure was used to find the optimal neurons from 1 to 20. Finally, when the number of neurons was 8, the MSE was the smallest (see Fig 4). The Elman network structure with neuron 8, 12 input variables and 1 output variable was constructed, and the maximum number of iterations was 2000 (the error drop of the network was shown in Fig 5). The fitting RMSE of ElmanNN was 0.37, and the fitting MAE was 0.27. Using the ElmanNN to predict the incidence of HB in Guangxi from January 2019 to August 2019, the prediction RMSE was 0.89 and the prediction MAE was 0.70.

### Comparison between ARIMA model and ElmanNN model

**Fig 6:**
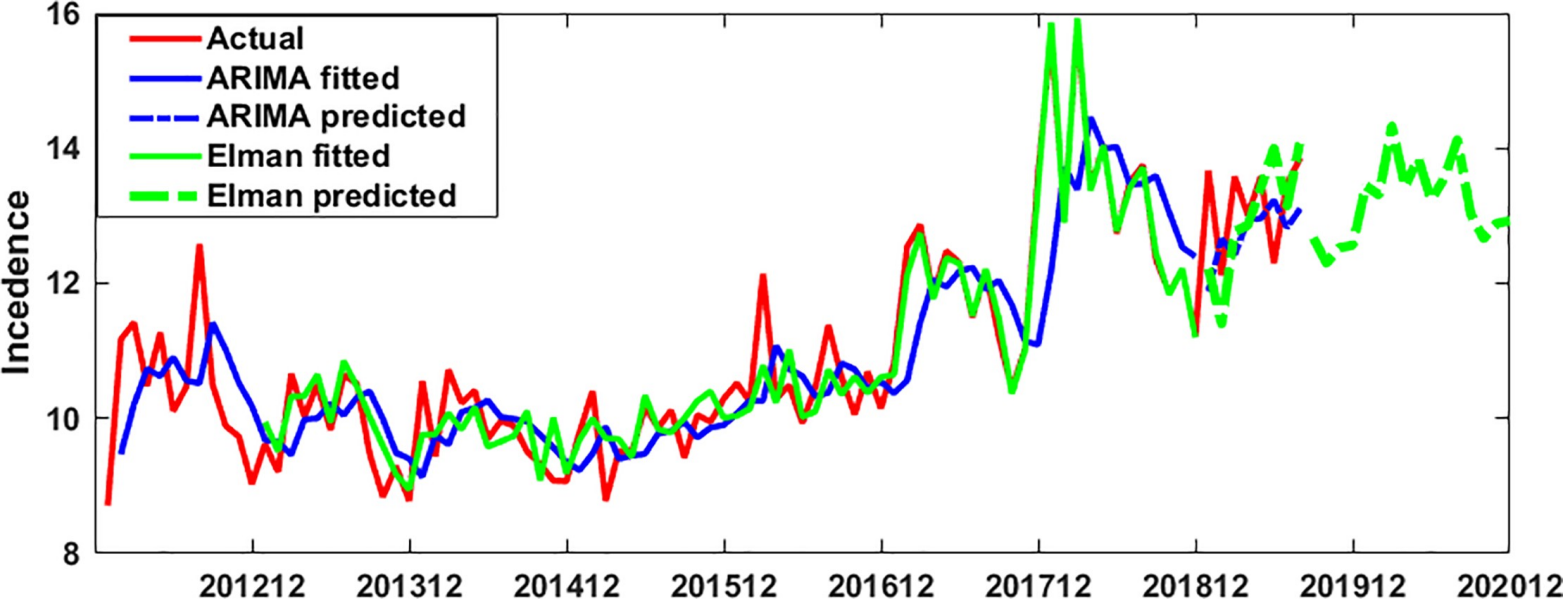
The fitting and prediction of HB incidence by ARIMA (0, 1, 1) and ElmanNN model.

RMSE and MAE indexes were used to compare ARIMA model with ElmanNN. The fitting and prediction index values of the two models were shown in Table 2. The fitting and prediction of the actual incidence of HB by the two models were shown in Fig 6.

**Table 2:** The fitting and prediction RMSE and MAE values of ARIMA (0, 1, 1) model and ElmanNN model.

	ARIMA(0,1,1)fitting	ARIMA(0,1,1)prediction	ElmanNN fitting	ElmanNN prediction
RMSE	0.95	0.94	0.37	0.89
MAE	0.7	0.81	0.27	0.70

## Discussion

Chronic hepatitis B, as a widely spread infectious disease, has brought great burden to the health cause all over the world. The World Health Organization has proposed that HB should be eliminated in 2030 [2]. China is the country with the heaviest burden of HB infection in the world. In order to achieve the goal of eliminating HB by 2030, it is necessary not only to do a good job in the treatment of HB, to improve the cure rate of HB, but also to do a good job in the prevention and control of HB. The incidence of HB in Guangxi is higher than that of the national level in China. It can be seen from the Fig 1 that the incidence of HB in Guangxi has shown an upward trend in recent years, which needs to be paid more attention to by the prevention and control departments, and analyzes the cause of increasing incidence and take necessary measures to prevent and control it. However, there was little research on the prediction of HB incidence in Guangxi at present; therefore, this study aimed to do prediction analysis of HB incidence in Guangxi by two widely popular prediction model methods, so as to provide scientific reference for prevention and control work.

The data of HB incidence from January 2012 to August 2019 were analyzed, the data from January 2012 to December 2018 were used as modeling data and the data from January 2019 to August 2019 as test model data.

First of all, we analyzed the feasibility of ARIMA model prediction. Through the data analysis of the modeling part, it was found that the data were stable after the first order difference. The possible p, q values in ARIMA (p, 1, q) were determined by drawing ACF and PACF maps on the stationary data. Finally, only the ARIMA (0, 1, 1) model passed the parameter test, and the residual of the model was also white noise, which indicated that the model has good performance and could be used to predict the incidence of HB in Guangxi. Using this model to predict the incidence data of HB in Guangxi from January 2019 to August 2019, the prediction RMSE was 0.94 and the prediction MAE was 0.81, the two values were relatively small, which indicated that the prediction accuracy of the model was high. Secondly, we analyzed the feasibility of ElmanNN prediction, and constructed the input and output matrix of ElmanNN model based on Guangxi HB incidence data from January 2012 to August 2019. When the number of neurons was 8, the network was optimal. We used the optimal ElmanNN network to fit and predict the HB incidence data in Guangxi,China, the prediction RMSE was 0.89, and the prediction MAE was 0.70, these two values were relatively small, which indicated that the prediction accuracy of the model was high. The prediction performance of ARIMA model and Elman model was compared by RMSE and MAE. From Table 2, it was found that the RMSE and MAE values of Elman model were smaller than those of ARIMA model, which indicated that Elman model had better prediction performance. Using ARIMA model and Elman model to fit the observed data of hepatitis B incidence (see Fig 6); we could see that the fitting effect of Elman model was better than that of ARIMA model, especially from December 2016 to December 2018. Based on the ElmanNN model, we made a prediction analysis of the HB incidence in Guangxi from September 2019 to December 2020(see Fig 6), and the forecast results showed that the trend of HB incidence in 2020 was similar to that in 2019 and slightly higher than that in 2019. According to the forecast results of this study, the department of disease prevention and control should make the optimal allocation of resources in advance, so as to better serve the health protection work of the HB.

## Conclusions

The World Health Organization has put forward the goal of eliminating hepatitis B in 2030. However, in recent years, the incidence of hepatitis B in Guangxi, China, has shown an upward trend, which needs to attract the attention of disease prevention and control departments, some departments should take joint actions to reduce the incidence of HB, and research from different angles should also be carried out. However, at present, there is little research on quantitative prediction of Hepatitis B in Guangxi. Therefore, in order to do a good job in prevention and resource allocation, it is necessary to give prediction and analysis in advance. Hence, this study used the widely used ARIMA model and Elman neural network to predict the incidence of HB in Guangxi. The results showed that ARIMA (0, 1, 1) model and ElmanNN model could be used to predict the incidence of HB in Guangxi, and the prediction performance was good, and the prediction accuracy of the ElmanNN model was higher than that of the ARIMA model. Based on the ElmanNN model, we predicted the HB incidence from September 2019 to December 2020, the predicted results showed that the incidence of HB in 2020 was slightly higher than that in 2019 and the change trend was similar to that in 2019, for 2021 and beyond, the ElmanNN model could be used to continue the predictive analysis of HB incidence in Guangxi,China.

At present, under the severe situation of rising HB incidence in Guangxi, some institutions need joint action to help do a good job in the prevention and control of HB. This study only made an analysis from the aspect of incidence prediction, and provided some reference for the optimal allocation of health resources in advance. Some studies had shown that many factors were related to the high incidence of diseases, such as socio-economic factors [34,35], demographic factors [36,37] meteorological factors [38–41], etc. The related research on the influencing factors of HB incidence should also be carried out in time, which can also help the prevention and control of HB. In further research work, we will use appropriate statistical methods to explore the key factors affecting HB incidence.

## Supporting information

S1 File Data set of HB incidence used in the manuscript, as shown in Fig 1. (XLSX)

S2 File The flowchart of Elman network establishment process. (TIF)
