# Supplementary figures and images for: A comparative study of two methods to predict the incidence of hepatitis B in Guangxi, China
Source: PLoS One. 2020 Jun 24;15(6):e0234660. doi: 10.1371/journal.pone.0234660 (PMC7314421; doi:10.1371/journal.pone.0234660)

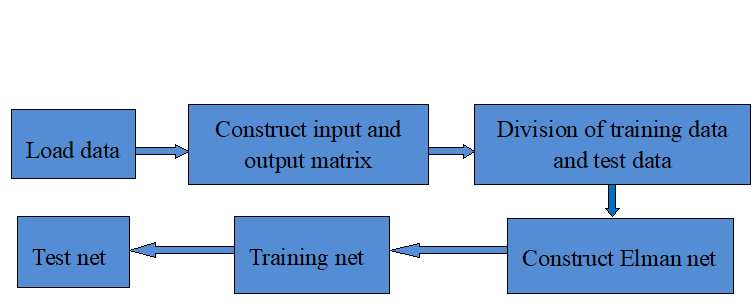

Supplement: S2 File — (TIF) [file pone.0234660.s002.tif]
